# Supplementary material for: A longitudinal study of risk factors associated with white spot disease occurrence in marine shrimp farming in Rayong, Thailand
Source: PeerJ. 2022 Mar 25;10:e13182. doi: 10.7717/peerj.13182 (PMC8958964; doi:10.7717/peerj.13182)
Supplement: Supplemental Information 2 [file peerj-10-13182-s002.docx]

**Appendix 1**

**A longitudinal study of risk factors associated with white spot disease occurrence in marine shrimp farming in Rayong, Thailand**

Sompit Yaemkasem, Visanu Boonyawiwat, Jiraporn Kasornchandra, Chaithep Poolkhet^*^

*Corresponding author: [fvetctp@ku.ac.th](mailto:fvetctp@ku.ac.th)

The questionnaire/structured interview schedule is a part of a research project of the Faculty of Veterinary Medicine, Kasetsart University.

Please note that the original questionnaire/structured interview schedule is in Thai

Questionnaire No………………. Date……………………

**1. General information**

1.1 Interviewer name……………………….….

1.2 Name of Responder…………………….…....................Gender ☐ Male ☐ Female

Role in farm……………………………………….…….

Farm name.................................

Address.........................................................................................................................

Tel:....................................

Pond identification (code/others)……………………………………………….…….

1.3 Farm coordinates………………………………………………………………….

1.4 Farm age……………………….years

1.5 Personnel who operates the farm (selecting more than one item is allowed)

☐ Yourself

☐ Workers

☐ Manager

☐ Others (please specify)………………….

1.6 Do you have other farms under your care?

☐ No

☐ Yes, (please specify)……………………

1.7 Species cultivation in this crop

☐ White pacific shrimp

☐ Black tiger shrimp

☐ Other (please specify)………………………………………………

1.8 How many crops do you produce per year in this farm?....................................................

1.9 Please specify, the cultivation of previous crop in this pond…………………….

……………………………………………………………………………………………

1.10 For this ponding, please acknowledge the following questions

Source of post-larvae (PL)……………………

Age of PL…………………….…

Price…………………………….

**2. Disease status**

2.1 The results of White spot disease (WSD) test by nested PCR (Author’s notes)

☐ Negative (Note; test date/place of test/others)

For PL…………………………………………………………

For shrimp…………………………………………………….

☐ Positive (Note; test date/place of test/others)

For PL…………………………………………………………

For shrimp…………………………………………………….

2.2 Age of shrimp.........................(days of stocking)

2.3 Did they have any signs of WSD? (Selecting more than one item is allowed)

☐ Reddish to pinkish discoloration

☐ Presence of white inclusion

☐ Other (please specify such as swimming pattern/others)

………………………………………………………………….…

2.4 Morbidity rate…….................

2.5 Mortality rate..........................

2.6 Did this farm develop WSD?

☐ No

☐ Yes (when/Laboratory Results/other)………………………………………………………….

2.7 Did this crop develop WSD previously?

☐ No

☐ Yes (when/Laboratory Results/others)………………………………………………………….

**3. Farm characteristic**

3.1 Total farm area……….(Rai) (Rai is a Thai unit of area equal to 1,600 square meters; Authors)

3.2 Area of ponding………...(Rai)

3.3 Total number of ponds.………………….

3.4 Number of active ponds…………………

3.5 Number of active ponds during this data collection……………….

3.6 Size of this pond……………………………

3.7 Stocking density of this pond.........................PL/m^2^

**4. Geographical factors**

4.1 Distance from the source of sea water (nearest border of farm)

……………………………………………(Kilometers/meters)

4.2 Distance from the source of sea water (nearest border of this pond)

……………………………………………(Kilometers/meters)

4.3 Distance from road (nearest border of farm)

……………………………………………(Kilometers/meters)

4.4 Distance from road (nearest border of this pond)

……………………………………………(Kilometers/meters)

4.5 Distance from another farm (nearest farm)

……………………………………………(Kilometers/meters)

4.6 Please describe the weather during this ponding

…………..,…………………………………………………………………………………

4.7 Do you think the weather in this area is rapidly changing and affects this ponding?

…………..,…………………………………………………………………………………

**5. Water management**

5.1 Do you have a reservoir pond?

☐ No

☐ Yes, please specify number of reservoir pond and size of each

.......................................................................................................

.......................................................................................................

5.2 Do you have a sludge pond?

☐ No

☐ Yes, please specify number of reservoir pond and size of each

.......................................................................................................

.......................................................................................................

5.3 What type of water managing system is in your farm?

☐ 100% recycle

☐ Partial recycle

☐ 100% Release

☐ Other (please specify)………………………………………………

5.4 What is the source of water for ponding? (Selecting more than one item is allowed)

☐ Sea

☐ Public canal

☐ Underground water

☐ River

☐ Rainfall

☐ Other (please specify)………………………………………………

5.5 In the preparation step of this pond, for water treatment, which of the following substance(s) did you apply (selecting more than one item is allowed)

☐ Trichlorfon (please specify the mode of application)………………………………………

☐ Copper sulfate (please specify the mode of application)…………………………………

☐ Tea seed cake (please specify the mode of application)……………………………………

☐ Chlorine (please specify the mode of application)…………………………………………

☐ Iodine (please specify the mode of application)………………………………………….…

☐ Probiotics (please specify the mode of application)…………………………………………

☐ Others (please specify)……………………………………………………………….

5.6 Please describe your method of pond preparation in this crop

………………………………………………………………………………………………………………………………………………………………………………………………………………………………………………………………………………………………

5.7 Did you add water during stocking period?

☐ No

☐ Yes, please specify the number of times and estimated volume……………...................

5.8 Did you retain the water before adding during the stocking period?

☐ No

☐ Yes, please specify the number of times and estimated volume……………...................

5.9 Please specify the source of the added water

☐ reservoir

☐ public canal

☐ sea directly

☐ Others (please specify)……………………………………………………………….

5.10 Did you treat the added water during ponding?

☐ No

☐ Yes, (please specify)…………………………………………………………………….

5.11 Did you use a probiotic during ponding?

☐ No

☐ Yes, (please specify)…………………………………………………………………….

5.12 Generally, after harvest, did you treat the cultured water before releasing it into a natural source?

☐ No

☐ Yes, (please specify)…………………………………………………………………….

5.13 During WSD outbreak in your area, did you treat the cultured water before releasing it into a natural source?

☐ No

☐ Yes, (please specify)…………………………………………………………………….

5.14 Do you have any other information regarding water management to prevent WSD? ………………………………………………………………………………………………………………………………………………………………………………………………

**6. Biosecurity measures**

6.1 Is the farm fully fenced?

☐ No

☐ Yes

☐ Others (please specify)……………………………………………………………….

6.2 Please describe a biosecurity measure on human movement

……………………………………………………………………………………….

……………………………………………………………………………………….

6.3 Please describe a biosecurity measure on vehicle movement

……………………………………………………………………………………….

……………………………………………………………………………………….

6.4 Do you have any freely-roaming pets in the farm?

☐ No

☐ Yes, please specify (what kind of pet/number of appearances per day/others)

……………….…………………………………………………...............................

6.5 Do you separate the work equipment for each pond?

☐ No

☐ Yes

☐ Others (please specify)……………………………………………………………….

6.6 Is every pond lined with a polyethylene sheet?

☐ No

☐ Partial (please described the method)……………………………………………

☐ Yes (please described the method)………………………………………………

☐ Others (please specify)……………………………………………………………….

6.7 In this pond, do you have the following biosecurity?

☐ None

☐ Bird-proof netting

☐ Crab-proof fencing

☐ Hand and foot disinfectant baths.

☐ Others (please specify)……………………………………………………………….

6.8 Is the water filtered through a trawling net before releasing into culture ponds?

☐ No

☐ Yes, type of filter………size of mesh............... number of layers..................

☐ Others (please specify)……………………………………………………………….

6.9 By observation of interviewer, please describe other biosecurity measure in each pond during this data collection

……………………………………………………………………………………….

……………………………………………………………………………………….

**7. Pond management (Focusing Pond)**

7.1 Was the sludge (soil at the bottom of the pond) removed in this pond before cultivation?

☐ No

☐ Yes

☐ Others (please specify)……………………………………………………………….

7.2 Do you plough the bottom up?

☐ No

☐ Yes…………………………….

☐ Others (please specify)……………………………………………………………….

7.3 Do you dry the pond before use?

☐ No

☐ Yes, how long………………………………………………….............................

☐ Others (please specify)……………………………………………………………….

7.4 Did you use lime for bottom-lining the pond?

☐ No

☐ Yes, how/concentration..........................................................................................

☐ Others (please specify)……………………………………………………………….

7.5 Did you use chicken/pig manure or cow dung to fertilize the pond?

☐ No

☐ Yes, how...............................................................................................................

☐ Others (please specify)……………………………………………………………….

7.6 Did you use inorganic fertilizer to adjust water coloring?

☐ No

☐ Yes, how...............................................................................................................

☐ Others (please specify)……………………………………………………………….

7.7 Did you use any antibiotic during ponding?

☐ No

☐ Yes, how/concentration..........................................................................................

☐ Other please specify………………………………………………………………

7.8 Please describe all methods applied by you after harvesting in this pond (if yes)

……………………………………………………………………………………….

……………………………………………………………………………………….

**8. Feeding management**

8.1 Please describe a source of pelleted feed/provider during this period (for all active ponds and how to apply in this pond/Did you use a multiple source of provider?)

……………………………………………………………………………………….

……………………………………………………………………………………….

……………………………………………………………………………………….

8.2 Did you use live feed?

☐ No

☐ Yes, how...............................................................................................................

☐ Others (please specify)……………………………………………………………….

8.3 Did you use fresh feed?

☐ No

☐ Yes, how...............................................................................................................

☐ Others (please specify)……………………………………………………………….

8.4 Did you check for WSD in live/fresh feed?

☐ No

☐ Yes, how...............................................................................................................

☐ Others (please specify)……………………………………………………………….

8.5 Please describe the feed supplementation used in this posing

……………………………………………………………………………………….

……………………………………………………………………………………….

……………………………………………………………………………………….

8.6 Did you use/mix any probiotic in pelleted feed?

☐ No

☐ Yes, type of probiotic/how.....................................................................................

☐ Others (please specify)……………………………………………………………….

8.7 Any other comments (ask the interviewee)

……………………………………………………………………………………….

……………………………………………………………………………………….

……………………………………………………………………………………….

……………………………………………………………………………………….

……………………………………………………………………………………….

……………………………………………………………………………………….

8.8 For interviewer, if you have any interesting finding associated with WSD in this farm, please note here;

……………………………………………………………………………………….

……………………………………………………………………………………….

……………………………………………………………………………………….

……………………………………………………………………………………….

……………………………………………………………………………………….

……………………………………………………………………………………….

*************
