# Supplementary material for: A longitudinal study of risk factors associated with white spot disease occurrence in marine shrimp farming in Rayong, Thailand
Source: PeerJ. 2022 Mar 25;10:e13182. doi: 10.7717/peerj.13182 (PMC8958964; doi:10.7717/peerj.13182)
Supplement: Supplemental Information 5 [file peerj-10-13182-s005.docx]

**แบบสอบถามงานวิจัย เรื่อง** **ศึกษาแนวทางการจัดการน้ำและพาหะ เพื่อลดปัญหาโรคตัวแดงดวงขาวที่เกิดโรคต่อเนื่องในฟาร์มเลี้ยงกุ้งทะเล ในจังหวัดระยอง**

**ผู้รับผิดชอบ: สมพิศ แย้มเกษม**

**โทร. 0897646781**

**วัตถุประสงค์ของโครงการ**

เพื่อหารูปแบบการจัดการน้ำ การจัดการฟาร์ม การเตรียมบ่อ ที่เหมาะสมเพื่อนำไปใช้ลดปัญหาการโรคตัวแดงดวงขาวในฟาร์มที่เคยเกิดเป็นประจำ

**ข้อควรพิจารณาหรือแจ้งเกษตรกร**

การศึกษาและรวบรวมข้อมูล วิธีการจัดการน้ำ การจัดการฟาร์ม และสัตว์ประจำถิ่นที่อาจเป็นพาหะนำโรคตัวแดงดวงขาวของฟาร์มที่เกิดโรคตัวแดงดวงขาวในรอบ 3 ปี ในเขตจังหวัดระยอง ตั้งแต่เดือนตุลาคม 2557 ถึงเดือนกันยายน 2560

คณะผู้วิจัยขอขอบพระคุณในความร่วมมือ

ข้อมูลของท่านจะถูกปกปิดไว้เป็นความลับและนำเสนอในภาพรวมเท่านั้น

**ข้อมูลทั่วไป**

1. วันที่ทำแบบสอบถาม_________________________
2. ชื่อและสกุลของผู้ให้ข้อมูล________________________________________
3. ตำแหน่งในฟาร์ม______________________________
4. ที่ตั้งฟาร์ม เลขที่______________หมู่ที่_________ชื่อหมู่บ้าน______________ตำบล_____________อำเภอ__________________จังหวัด___________________โทรศัพท์_______________________ พิกัด GPS __________________________
5. ชื่อฟาร์ม________________________
6. อายุฟาร์ม__________________________
7. ผู้ที่มีส่วนเกี่ยวข้องกับการเลี้ยง เช่น การให้อาหาร การให้สารเสริม การจัดการน้ำ และอื่นๆ (เลือกได้มากกว่า 1 ข้อ)

เจ้าของ ผู้จัดการ คนงาน ผู้อื่น (ระบุ)______________

1. เจ้าของฟาร์มหรือผู้จัดการมีฟาร์มในความดูแลกุ้งกี่ฟาร์ม

 1 ฟาร์ม  มากกว่า 1 ฟาร์ม

1. ใน 1 ปีเลี้ยงกุ้งกี่รอบ ________________________

**ข้อมูลการเกิดโรค**

1. โปรดวาดแผนผังของฟาร์ม โดยระบุถนน แหล่งน้ำ รวมถึงตำแหน่งของบ่อในฟาร์ม พร้อมระบุบ่อที่เป็นโรค WSD (หากมี) และ/หรือบ่อที่ไม่เป็นโรค รวมถึงตำแหน่งเกิดโรคข้างๆ

2. ฟาร์มที่กำลังสอบถาม มีบ่อที่เป็น case หรือ control (อย่างใดอย่างหนึ่ง)

2.1 case (พบโรค WSD)

เป็นบ่อหมายเลข_____________________

ขนาดบ่อ___________________

จำนวนปล่อย___________________

ลูกกุ้งจากบริษัทไหน___________________

ในช่วงเวลาดังกล่าวพบปัญหาดังต่อไปนี้หรือไม่

การตายผิดปกติ

พบที่อายุกุ้ง______________วัน

การตายสะสมเป็นกี่เปอร์เซ็นต์ในช่วง 3-5 วันแรก_________%

ลักษณะของกุ้งที่พบ (เลือกได้หลายข้อ)

ตัวแดง มีจุดขาว อื่นๆ_______________

อาการของกุ้งที่พบ

แตะขอบบ่อ

ว่ายควงสว่าน

ตายจม/ตายในยอ

อื่นๆ___________________

**ลักษณะการกินอาหารผิดปกติหรือไม่**  ไม่ น้อยลง มากขึ้น

เลี้ยงต่อหรือไม่__________________________________

จัดการน้ำ กุ้งหลังเกิดโรคอย่างไร_________________________________________________

2.2 control (ไม่พบโรค WSD)

เป็นบ่อหมายเลข_____________________

ขนาดบ่อ___________________

จำนวนปล่อย___________________

ลูกกุ้งจากบริษัทไหน___________________

3. การตรวจยืนยันการเกิดโรค

3.1 WSSV strip test

 เป็น  ไม่เป็น

3.2 PCR

 เป็น  ไม่เป็น

4. วันที่เกิดโรค ___________________

5. ชนิดกุ้งที่เลี้ยง

กุลาดำ กุ้งขาวแวนนาไม

6. รุ่นก่อนหน้าฟาร์มเคยมีกุ้งป่วยด้วยโรคตัวแดงดวงขาวหรือไม่

 เป็น ___________________  ไม่เป็น

**ข้อมูลฟาร์ม**

1. พื้นที่ฟาร์ม ___________________

2. พื้นที่เลี้ยง ___________________

3. จำนวนบ่อทั้งหมด_____บ่อ จำนวนบ่อที่เลี้ยงจริง____บ่อ ณเวลานั้น

4. อัตราการปล่อย ___________________

5. แหล่งลูกพันธุ์ ___________________ อายุ ___________________ ราคา __________________

6. ขนาดบ่อเลี้ยง ___________________

**ปัจจัยแวดล้อมอื่นๆ**

1. ระยะห่างจากฟาร์มกับแหล่งน้ำธรรมชาติ ___________________

2. ระยะห่างจากฟาร์มกับถนนสายหลัก ___________________

3. ฟาร์มข้างเคียงระยะห่าง 1 กิโลเมตร

 ไม่มี  มี ___________________

4. ช่วงที่เลี้ยงมีสภาพอากาศแปรปรวนหรือไม่

 ไม่มี  มี ___________________

5. ระหว่างการเลี้ยงน้ำในบ่อแพลงก์ตอนดรอปหรือสีน้ำล้มหรือไม่

 ไม่มี  มี ___________________

**การจัดการน้ำ**

1. มีบ่อพักน้ำหรือไม่

 ไม่มี  มี ขนาด ___________________ ไร่

2. ฟาร์มมีบ่อเก็บเลนหรือไม่

 ไม่มี  มี ขนาด ___________________ ไร่

3. ฟาร์มนำน้ำกลับมาให้ใหม่หรือไม่

100% นำกลับมาใช้ใหม่

นำกลับมาใช้บางส่วน

ปล่อยทิ้งทั้งหมด

4. ฟาร์มใช้น้ำจากแหล่งน้ำใดในการเลี้ยงกุ้ง

ทะเล คลองสาธารณะ น้ำบาดาล แม่น้ำ น้ำฝน

**ระบบความปลอดภัยทางชีวภาพ**

1. ฟาร์มมีรั้วหรือไม่ (คลองกั้นอาณาเขต)

 ไม่มี  มี ___________________

2. ฟาร์มมีการห้ามบุคคลภายนอกเข้าออกหรือไม่

 ไม่มี  มี ___________________

3.ระหว่างการเลี้ยงคนงานเข้า-ออกจากฟาร์มหรือไม่

 ไม่มี  มี ___________________ครั้ง

4. ฟาร์มปล่อยสัตว์เลี้ยงให้สามารถวิ่งเล่นอยู่ในฟาร์มหรือไม่

 ไม่มี  มี ___________________

5. ฟาร์มมีการใช้สารฆ่าเชื้อป้องกันเชื้อโรคที่มากับรถยนต์หรือไม่

ใช้เครื่องสเปรย์ อ่างล้างล้อ ไม่มี

6. ฟาร์มมีที่ล้างมือ-เท้า หรือห้องอาบน้ำเพื่อฆ่าเชื้อสำหรับคนที่จะเข้าฟาร์มหรือไม่

 ไม่มี  มี ___________________

7. ฟาร์มมีการแยกคนงานและอุปกรณ์สำหรับแต่ละบ่อหรือโซนหรือไม่

 ไม่มี  มี ___________________

8. ฟาร์มมีการปูพลาสติก PE รอบบ่อหรือไม่

ทั้งบ่อ เฉพาะ slope ไม่มี

9. ฟาร์มมีระบบความปลอดภัยทางชีวภาพเหล่านี้หรือไม่

เชือกกันนก รั้วกันปู อ่างล้างมือล้างเท้าฆ่าเชื้อ ไม่มี

10. ลูกกุ้งระยะ PL มีการตรวจไวรัสตัวแดงดวงขาวก่อนปล่อยหรือไม่

 ไม่มี  มี ___________________

**การเตรียมบ่อ**

1. ฟาร์มมีการนำเลนก้นบ่อออกทุกรอบการเลี้ยงหรือไม่

 ไม่มี  มี ___________________

2. ฟาร์มมีการไถพรวนพื้นบ่อหรือไม่

 ไม่มี  มี ___________________

3. ฟาร์มมีการตากบ่อก่อนใช้หรือไม่

 ไม่มี  มี ,ตาก ___________________ วัน

4. ฟาร์มมีการใช้ปูนขาวพื้นบ่อหรือไม่

 มี ___________________ กิโลกรัม/ไร่

ไม่มี อธิบายวิธีการ ___________________

5. ฟาร์มมีการใช้ยาปฏิชีวนะระหว่างการเลี้ยงหรือไม่

 ไม่มี  มี ชนิดยา ___________________ โดสที่ใช้ ___________________

6. ฟาร์มมีการใช้จุลินทรีย์ก่อนเตรียมน้ำหรือไม่

 ไม่มี  มี ชนิด ___________________ ปริมาณที่ใช้ ___________________

7. ฟาร์มมีการใช้จุลินทรีย์ระหว่างการเลี้ยงหรือไม่

 ไม่มี  มี ชนิด ___________________ ปริมาณที่ใช้ ___________________

8. ฟาร์มมีการใช้วิตามินระหว่างการเลี้ยงหรือไม่

 ไม่มี  มี ชนิด ___________________ ปริมาณที่ใช้ ___________________

**การเตรียมน้ำ**

**1**. การฆ่าเชื้อในน้ำ สารเคมีที่ใช้

ไตรคลอฟอน คอปเปอร์ซัลเฟต กากชา คลอรีน

ไอโอดีน โปรไบโอติก อื่นๆ ___________________

ไตรคลอฟอน คอปเปอร์ซัลเฟต กากชา คลอรีน

ไอโอดีน โปรไบโอติก อื่นๆ ___________________

2. การกรองน้ำก่อนเข้าบ่อ

กรอง ชนิดผ้ากรอง ___________________ ขนาดผ้ากรอง ___________________

ผ้ากรองกี่ชั้น ___________________

 ไม่มี

3. ฟาร์มมีใช้ปุ๋ยมูลสัตว์ลงบ่อหรือไม่

 ไม่มี  มี ขนาดที่ใส่ ___________________

4. ฟาร์มมีการใช้ปุ๋ยวิทยาศาสตร์หรือไม่

 ไม่มี  มี ขนาดที่ใส่ ___________________

5. อธิบายวิธีการเตรียมน้ำสำหรับเลี้ยงกุ้งของฟาร์ม

..................................................................................................................................................................................................................................................................................................................................................................................................................................................................................................................................................................................................................................................................................................................................................................................................................................................................................................................................................................

6. วิธีการเตรียมน้ำก่อนเลี้ยงเป็นรูปแบบไหน

6.1 พัก วันแล้วสูบเข้าบ่อเลี้ยงเลยไม่มีการฆ่าเชื้อ

6.2 พัก วันแล้ว treatด้วย ในบ่อพักแล้วสูบลงบ่อเลี้ยง

6.3 พัก วันแล้วสูบลงบ่อเลี้ยง treatด้วย ในบ่อเลี้ยง

6.4 พัก วันแล้วสูบไปบ่อ treatด้วย ในบ่อ treat ปั๊มไปบ่อชงจากนั้นไปลงบ่อเลี้ยง

6.5 อื่นๆ

การเตรียมน้ำระหว่างเลี้ยง

1.. ฟาร์มมีการเติมน้ำระหว่างเลี้ยงหรือไม่

 ไม่มี  มี จำนวน ___________________ ครั้ง

2. ฟาร์มมีการพักน้ำก่อนการเติมน้ำระหว่างเลี้ยงหรือไม่

 ไม่มี  มี

3. ระยะเวลาในการพัก ___________________ วัน

3. แหล่งน้ำที่ใช้เติมมาจากที่ใด

 บ่อพักน้ำ  คลองหรือทะเลโดยตรง

4. น้ำที่เติมระหว่างการเลี้ยงมีการฆ่าเชื้อหรือไม่

 ไม่มี  มี

5. ฟาร์มฆ่าเชื้อน้ำที่เติมระหว่างการเลี้ยงก่อนหรือไม่

ไตรคลอฟอน___________________ ppm. กากชา ___________________

คลอรีน ___________________ ไอโอดีน ___________________

อื่นๆ ___________________ ไม่ฆ่าเชื้อ

**การจัดการน้ำหลังการเลี้ยง**

1. มีการฆ่าเชื้อก่อนปล่อยออกหรือไม่

 ไม่มี  มี

2.สารเคมีที่ใช้ในการฆ่าเชื้อ

ไตรคลอฟอน___________________ ppm. คลอรีน ___________________

ไอโอดีน ___________________

อื่นๆ ___________________

3.อธิบายวิธีการจัดการน้ำหลังการเลี้ยง

........................................................................................................................................................................................................................................................................................................................................................................................................................................................................................................................................................................................................................................................................................................................

**การจัดการน้ำออกในระหว่างการเลี้ยง**

1.มีการดูดน้ำออกระหว่างการเลี้ยงหรือไม่

 ไม่มี  มี

2.กรณีมีการดูดน้ำออกทำอย่างไร

ดูดน้ำออกทุกวันและปล่อยลงคลองสาธารณะ

ดูดน้ำออกทุกวันปล่อยลงบ่อทิ้งเลนรวมของตัวเอง

ไม่ดูดน้ำออก

อื่นๆ ___________________

**การจัดการอาหาร**

1. อาหารที่ให้

อาหารสำเร็จรูป ยี่ห้อ___________________ อาหารมีชีวิต ___________________

อาหารสด ___________________

2. ฟาร์มมีการตรวจโรคตัวแดงดวงขาวในอาหารสดหรือไม่

 ไม่ตรวจ  ตรวจ ___________________

3.ฟาร์มผสมโปรไบโอติกในอาหารให้กุ้งกินหรือไม่

ใช่ ชนิดของโปรไบโอติก___________________ วิธีการใช้ ___________________

ไม่ผสม

ข้อคิดเห็นอื่นของเกษตรกรต่อสาเหตุการเกิดโรคตัวแดงดวงขาว

..................................................................................................................................................................................................................................................................................................................................................................................................................................................................................................................................................................................................................................................................................................................................................................................................................................................................................................................................................................

อธิบายวิธีการเลี้ยงและการจัดการฟาร์มคร่าว พอสังเขป เช่น เลี้ยงกี่วัน มีการอนุบาลก่อนปล่อยกี่วัน หรือปล่อยตรงเลยหรือไม่ เทคนิคที่ตัวเองคิดว่าทำให้สามารถปลอดโรคได้........................................................................................................................................................................................................................................................................................................................................................................................................................................................................................................................................................................................................................................................................................................................................................................................................................................................................................................................................................................................................................................................................................................................................................................................................................................................................................

***********************
